# The largest multicentre data collection on prepectoral breast reconstruction: The iBAG study

**DOI:** 10.1002/jso.26073

**Published:** 2020-08-12

**Authors:** Jaume Masià, iBAG Working Group, Marzia Salgarello, Leonardo Cattelani, Pier Camillo Parodi, Diego Ribuffo, Maria Giuseppina Onesti, Giorgio Berna, Simon J. Cawthorn, Fernando Bozza, Stefano Duodeci, Simon Harries, Maurizio Governa, Roberto A. Barmasse, Raghavan Vidya, Tapan Sircar, Harleen Deol, Tommaso Guzzetti, Fabrizio Meggiolaro, Mauro Schiavon, Rishikesh Parmeshwar, Douglas Ferguson, Caroline Mortimer, Giorgio Manca, Dinesh Thekkinkattil, Rathinasabapathy Rathinaezhil, Pud Bhaskar, Nicola Roche, Alberto Rivarossa, Gonzalo De Castro Parga, Samuele Massarut, Susanna Polotto, Mattia Di Bartolomeo, Emanuele Rampino Cordaro, Sebastiano Mura, Liliana Barone Adesi, Giovanni Marruzzo, Francesco Dell'Antonia, Monia Maritan, Tania Saibene, Silvia Michieletto, Claudia Cecconi, Manuele Maino, Dayalan Clarke, Sara Mirandola, Lucia Morelli, Mario Biral, Roberto Baraziol, Chiara Zanin, Chiara Gregorelli

**Affiliations:** ^1^ Department of Plastic Surgery, Hospital de la Santa Creu i Sant Pau Universitat Autonoma de Barcelona Barcelona Spain; ^2^ Department of Plastic Surgery and Reconstructive Surgery Fondazione Policlinico Universitario Agostino Gemelli IRCCS, Università Cattolica del Sacro Cuore Rome IT; ^3^ Breast Surgery Unit University Hospital of Parma Parma IT; ^4^ Department of Plastic and Reconstructive Surgery Azienda Ospedaliero‐Universitaria “Santa Maria della Misericordia” Udine IT; ^5^ Plastic Surgery Unit, Department of Surgery "P. Valdoni" Sapienza University Rome IT; ^6^ Plastic and Reconstructive Surgery Department, “Ca’ Foncello” General Hospital AULSS2 Marca Trevigiana Treviso IT; ^7^ Southmead Hospital Bristol North Bristol NHS Trust Bristol UK; ^8^ Breast Care Center IOV Istituto Oncologico Veneto ‐ IRCCS Padova IT; ^9^ Breast Unit, Hospital of Cittadella AULSS6 Euganea Padova IT; ^10^ Warwick Hospital South Warwickshire NHS Foundation Trust Warwick UK; ^11^ Department of Plastic and Reconstructive Surgery Azienda Ospedaliera Universitaria Integrata Verona IT; ^12^ Unit of Thoracic and Breast Surgery “Umberto Parini” General Hospital Aosta IT; ^13^ New Cross Hospital Royal Wolverhampton NHS Trust Wolverhampton UK; ^14^ East and North Hertfordshire NHS Trust Welwyn Garden City UK; ^15^ Plastic Surgery Unit ASST "Alessandro Manzoni" Hospital Lecco IT; ^16^ Breast Unit, Mirano Hospital AUSSL3 Serenissima Venezia IT; ^17^ Department of Breast and General Surgery University Hospitals of Morecambe Bay NHS Foundation Trust Lancaster UK; ^18^ Royal Devon and Exeter Hospital Royal Devon and Exeter NHS Foundation Trust Exeter UK; ^19^ Ipswich Hospital East Suffolk and North Essex NHS Foundation Trust Ipswich UK; ^20^ Department of Plastic Surgery, Spedali Civili Brescia University of Brescia Brescia IT; ^21^ Department of Oncoplastic Breast Surgery, Pilgrim Hospital and Lincoln County Hospital United Lincolnshire Hospitals NHS Trust Lincoln UK; ^22^ Park Centre for Breast Care Brighton and Sussex University Hospitals NHS Trust Brighton UK; ^23^ University Hospital of North Tees, North Tees and Hartlepool NHS Foundation Trust Stockton‐on‐Tees UK; ^24^ The Royal Marsden Hospital The Royal Marsden NHS Foundation Trust Chelsea, London UK; ^25^ Department of Plastic and Reconstructive Surgery “Santa Croce e Carle” Hospital Cuneo IT; ^26^ Breast Surgery University Hospital Complex of Vigo CHUVI Vigo ES; ^27^ Breast Surgery Unit CRO National Cancer Institute Aviano IT; ^28^ Plastic Surgery Unit, Department of Surgery “P. Valdoni” Sapienza University Rome IT

**Keywords:** acellular dermal matrix, braxon, breast reconstruction, prepectoral, radiotherapy

## Abstract

**Background and Objectives:**

In the last years, prepectoral breast reconstruction has increased its popularity, becoming a standard reconstructive technique by preserving pectoralis major anatomy and functionality. Nevertheless, the lack of solid and extensive data negatively impacts on surgeons’ correct information about postoperative complication rates and proper patient selection. This study aims to collect the largest evidence on this procedure.

**Methods:**

A multicentre retrospective audit, promoted by the Barcelona Hospital, collected the experience of 30 centers on prepectoral breast reconstruction with Braxon ADM. The study had the scientific support of INPECS and IIB societies which provided the online database Clinapsis.

**Results:**

A total of 1450 procedures were retrospectively collected in a 6‐year period. Mean age 52.4 years, BMI 23.9, follow‐up 22.7 months. Reconstruction was carried out after a tumor in 77.1% of the cases, 20.1% had prophylactic surgery, 2.8% had revisions. Diabetes, smoke, and immunosuppression had an influence on complications occurrence, as well as implant weight. Capsular contracture was associated with postoperative radiotherapy, but the overall rate was low (2.1%). Complications led to implant loss in 6.5% of the cases.

**Conclusions:**

The international Braxon Audit Group multicentre data collection represents a milestone in the field of breast reconstruction, extensively improving the knowledge on this procedure.

**Abbreviations**
ACCIb: Ibero‐American Cochrane Collaboration Association
ADM: acellular dermal matrix
BMI: body mass index
DTI: direct‐to‐implant
GAM: generalized additive models
iBAG: International Braxon Audit Group
IIB: Institut d'investigaciò Biomèdica Sant Pau
INPECS: Instituto Para la Excelencia Clínica y Sanitaria
PPBR: prepectoral breast reconstruction
RR: relative risk
RT: radiation therapy or radiotherapy

## 1 INTRODUCTION

Breast cancer nowadays represents the most common tumor among women, with over 2 million new cases in 2018. Currently, its surgical treatment is based on oncoplastic surgery, a synthesis between those two disciplines with the final goal of an outcome balanced between the best oncologic and cosmetic results. Indeed, since its conception, breast reconstruction helped patients look “normal” when dressed; more recently, advancements in surgical techniques and medical technologies have raised the bar so that patients can feel esthetically pleasing also unclothed.^1^ During the past decades, the surgical approach to breast cancer has evolved from radical mastectomy to the development of breast‐conserving surgery and reconstructive techniques. In fact, after a nipple or skin‐sparing mastectomy, the opportunity of having performed an immediate reconstruction represents a great advantage for patients, owing to its significant psychosocial benefits. Currently, immediate implant‐based breast reconstruction (IBR) represents 81.9% of all breast reconstructive procedures.^2^

However, only in the last decade, surgeons approached the prepectoral technique considering the importance of the role of the pectoralis major muscle and the possibility to avoid its recruitment for implant coverage: starting with the first cases described by Berna et al, the subcutaneous breast implant positioning becomes a concrete option, along with a complete breast implant wrapping with a large sheet of ADM to prevent the direct contact of the silicone prosthesis with the surrounding tissues.^3^, ^4^

Prepectoral breast reconstruction (PPBR) has quickly become more and more popular due to its satisfying esthetic outcomes, low functional detriment, and low complication rate. In fact, whereas the traditional submuscular reconstructive technique involves several drawbacks, such as unpleasant esthetic results and postoperative discomfort due to implant displacement, animation deformity, and capsular contracture, PPBR has overcome most of them.^5^, ^6^

Unfortunately, despite the current multiplicity of different devices committed to PPBR, there is still a lack of high‐quality evidence to support the safety of these new materials. In fact, most of the published studies are limited to small single‐center experiences with no homogeneous outcomes.^7^, ^8^, ^9^ As mentioned above, the first biologic membrane able to completely cover a silicone implant to position it subcutaneously was a 0.6 mm‐thick preshaped porcine ADM (Braxon; DECOmed Srl, Venice, Italy).^3^ To date, this device represents the most widely used one for PPBR in European and UK breast centers and the only ADM with a specific patented design that allows a standardized wrapping technique, reducing the number of variables so that data can be more easily compared among different centers.^10^ After a preliminary multicentre study on 100 PPBR cases carried out with Braxon ADM,^11^ the first author encouraged a larger EU‐UK database on PPBR performed with the same device to obtain valuable evidence about outcomes and risk factors potentially linked to postoperative complications: for this reason, the group of study was defined as international Braxon Audit Group (iBAG). Our study wants to represent an extensive and homogeneous retrospective multicentre data collection that sets the basis for future randomized prospective studies.

## 2 MATERIALS AND METHODS

### 2.1 Study design and participants

At the beginning of 2018 the coordinating center, Santa Creu i Sant Pau Hospital of Barcelona (Spain), promoted an international audit on PPBR to collect and analyze homogeneous data with the aim of creating the largest evidence on this procedure. This study had the scientific support of Institut d'investigaciò Biomèdica Sant Pau (IIB) and Instituto Para la Excelencia Clínica y Sanitaria (INPECS), created by the Ibero‐American Cochrane Collaboration Association (ACCIb) which provided the online database CLINAPSIS. The access to the database was protected by single‐user login credentials; it collected all information recorded in an anonymized format. Data were managed by the above‐mentioned independent organisms (IIB and INPECS) to avoid any bias.

The coordinating center of Barcelona invited to take part in the study of all EU and UK plastic and breast units performing PPBRs routinely and having performed more than 10 procedures with the Braxon ADM (DECOmed Srl). This collagen membrane is the most used for PPBR and the sole with a specific patented design that allows a standardized wrapping technique. The various options in ADM assembly constitute a critical point in the analysis of the outcomes of the procedure since the wrapping phase of the reconstructive technique is operator sensitive. The use of different devices to cover the breast implant could lead to inhomogeneous data collection due to the different materials and the nonstandardized implant covering procedure.

With the aim of obtaining uniform data, all the consecutive PPBRs carried out only with Braxon porcine ADM from November 2012 to June 2018 were retrospectively analyzed. Patients’ inclusion criteria to PPBR were defined according to each center practice; indeed, some general guidelines were already published about patient's selection for PPBR procedure and they include women with no history of radiation, non‐ or ex‐smokers, a good subcutaneous layer, a well‐perfused skin flap, an estimated mastectomy weight of less than 500 g and tumors not invading skin or chest wall, or those with inflammatory nature. Considering the retrospective nature of this study, the authors do not set any exclusion criteria with the aim to verify any association between patient characteristics and postoperative complications. Thus, if some of the 30 centers involved in the iBAG study enlarged the indication, therefore offering PPBR to thin or obese women, as well as diabetics or smokers, data coming from these cases were analyzed as well. The sole patients excluded from the analysis were those with less than 3 months of follow‐up.

Data analyzed were patients’ age, body mass index (BMI), comorbidities, smoking status, tumor classification, neo‐adjuvant or adjuvant chemotherapy, previous or adjuvant radiation therapy (RT), previous breast surgery, uni‐ or bilateral procedures, type of surgery (therapeutic, prophylactic or revision), type of mastectomy, breast skin incision, type of implant used, implant volume, axillary surgery, postoperative therapies, and complications. Complications were classified in “immediate” or “late” whether if they occurred respectively within or after 3 months from surgery. The complications observed included seroma, wound dehiscence, hematoma, skin necrosis, infection, implant extrusion, implant rotation, capsular contracture, rippling, and implant loss. Moreover, we collected all details about pre‐ and postoperative medical treatments, hospitalization period, and pre‐ and postoperative patients’ pictures.

### 2.2 Ethical standards

The research protocol of the study was approved by the Ethics Committee of the Santa Creu i Sant Pau Hospital; each participating center was required to obtain local committee approval. In accordance with the Declaration of Helsinki of 1975, as revised in 1983, all patients were asked to provide written consent for the use of their personal data, as part of the standard surgical consent of their institutions. Patients’ data were visible only to the supervisors of each center where the reconstructive procedure was carried out; no one else had access to those records. At the end of the period of data collection, the coordinating center had the authorization to manage all the anonymized data and to analyze them through statistical protocols.

### 2.3 Material and surgical procedure

**Figure 1:**
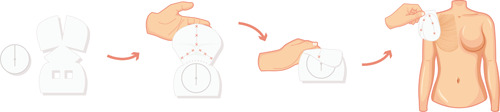
Standardized wrapping technique of Braxon ADM around the breast implant. The patented shaped allows a complete cover acting as an internal “implant bag” which distributes the weight of the silicone prosthesis around all the breast pocket and hides it from the direct contact with the organism. ADM acellular dermis matrix [Color figure can be viewed at wileyonlinelibrary.com]

After mastectomy or surgery performed for revision purposes, the reconstructive phase involves the preparation of the ADM, which requires rehydration of 5 minutes in room‐temperature saline solution to facilitate its handling. Then, the ADM is wrapped around the breast implant, its excess is cut away and it is closed by mean of absorbable 3/0 suture (Figure 1). Then, the completely wrapped implant is placed inside the breast pocket and fixed with cardinal stitches to the pectoralis major muscle fascia and to the subcutaneous layer through quilting sutures in the anterior part of the Braxon ADM, thus eliminating dead spaces and supporting ADM integration.

Surgeons involved in the study performed mastectomies according to specific clinical scenarios and their routine practice with the only common characteristic that the reconstructive phase did not entail the pectoralis major; implant selection (round or anatomical shape, fixed‐volume or adjustable implant, as well as temporary tissue expander) depended on surgeons’ preference. Breast reconstruction was then classified as two‐stage when performed with an expander or one‐stage when a definitive implant was used. In some cases, the ADM was used for patients requiring revision surgery due to implant‐related complications or undesired surgical outcomes.

In all cases, perioperative and postoperative antibiotics and drains were used according to local policy.

### 2.4 Statistical analysis

Considering the relevant sample size, *χ*² and the Student *t* test tests were firstly used to study all independent variables potentially influencing the studied outcome, particularly postoperative complications. A model was then designed for all variables to explain the evidence derived from the data set; it was based on hierarchical and generalized additive models (GAM), mainly dictated by the poor plausibility of a linear link between regressors and the response variable, which need to exploit the flexibility of the GAM models. Moreover, for each variable there were differences between several sample units dictated by some specific characteristics of the data collection, thus motivating the use of hierarchical models. Variables with more than 20% of missing data were excluded from the statistical analysis.

The adopted approach studies simple models first, with a single explanatory variable. After verifying the ability of these variables into the model description, they are included in a multiple logistic regression model, thus increasing the improvement of the estimates and the goodness of adaptation of the model to the data (Stepwise Method). The level for statistical significance was set at *P* < .05. All statistical analyses were performed with R version 3.5.1 (2018), Rstudio Version 1.1.456, CRAN project.

## 3 RESULTS

Thirty clinical centers, shared between Spain, UK, and Italy, took part in the study. Data collection started in September 2018 and finished in January 2019; preliminary data of the present study were presented at Barcelona Breast Meeting (BBM) and at Oncoplastic and Reconstructive Breast Surgery (ORBS) conference, both held in 2019.

The iBAG identified 1450 PPBR, carried out in 1186 women, in a 6‐year period comprised between November 2012 and June 2018. Demographic, clinical, and surgical details of the patients are explained in Table 1.

**Table 1:** Demographic, clinical and surgical details of the iBAG data collection

Details		No. (range/percentage)
No. patients		1186
No. breasts		1450
Follow‐up		22.7 ± 13.1 (3‐75.7)
Demographic/clinical		
AGE		52.4 ± 11.4 (17‐87)
BMI		23.9 ± 4.1 (15.6‐40)
Comorbidities	Obese	56 (4.7)
	Diabetes	28 (2.4)
	Vascular disease	12 (1.0)
	Hypertension	132 (11.1)
	Hypothyroidism	78 (6.6)
	Other not defined comorbidities	190 (16.0)
Smoking status	Active smokers	120 (10.1)
	Ex‐smokers	62 (5.2)
Use of drugs	Immunosuppressants	7 (0.6)
	Anticoagulants	26 (2.2)
	Use of other not defined drugs	197 (16.6)
Previous surgery (per breast)	Quadrantectomy	81 (5.6)
	Lumpectomy	58 (4.0)
	Mastectomy	18 (1.2)
	Reconstruction	24 (1.7)
	Esthetic surgery	32 (2.2)
Type of tumor (per breast)	DCIS	256 (17.7)
	LCIS	14 (1.0)
	IDC	623 (43.0)
	ILC	100 (6.9)
	Multiples /mixed	77 (5.3)
	Other	48 (3.3)
	None	298 (20.6)
Chemotherapy^a^	Neoadjuvant	141 (11.9)
	Adjuvant	216 (18.2)
	Both treatments	34 (2.9)
Radiotherapy^b^ (per breast)	Preoperative	42 (2.9)
	Postoperative	153 (10.6)
	Both treatments	3 (0.2)
Surgical (per breast)		
Type of surgery	Therapeutic	1118 (77.1)
	Prophylactic	291 (20.1)
	Revision	41 (2.8)
Axillary dissection		187 (12.9)
Mastectomies	Nipple‐sparing	720 (49.7)
	Skin‐sparing	466 (32.1)
	Skin‐reducing (NAC removal)	81 (5.6)
	Skin‐reducing (NAC preservation)	19 (1.3)
	None	35 (2.4)
	Not defined	129 (8.9)
Mastectomy weight		369.7 ± 201.4 (42‐1200)
Breast implant size (g)		349.2 ± 112.6 (100‐685)
Breast implant profile^c^	Round	125 (8.6)
	Anatomical	1298 (89.5)
Breast implant brand	Allergan	592 (40.8)
	Mentor	390 (26.9)
	Polytech	133 (9.2)
	Nagor	107 (7.4)
	Sebbin	15 (1.0)
	B‐lite	4 (0.3)
	Silimed	1 (0.1)
	Motiva	1 (0.1)
	Not defined	182 (12.6)
	Expander	25 (1.7)
Incisions	Inverted‐T	109 (7.5)
	Round block	29 (2.0)
	Inframammary fold	183 (12.6)
	Lateral	609 (42.0)
	Periareolar	99 (6.8)
	Elliptic	271 (18.7)
	Other	73 (5.0)
	Not defined	77 (5.31)
Hospital stay, d		3.2 ± 2.7 (0.5‐21.0)

Abbreviations: iBAG, International Braxon Audit Group; DCIS, ductal carcinoma in situi; IDC, invasive ductal carcinoma; ILC, invasive lobular carcinoma; LCIS, lobular carcinoma in situ; NAC, nipple‐areolar complex; RBS, red breast syndrome.

a Nineteen patients had chemotherapy but missing data on administration time.

b Nine breasts had missing data about radiation treatments.

c Twenty seven procedures had missing data on implant profile.

A *t*‐test analysis revealed a significant difference in hospitalization time mean for patients with hypothyroidism (*P*‐value = .016), with preoperative chemotherapy (*P*‐value = .004), patients who underwent axillary surgery (*P*‐value = .008), with postoperative chemotherapy (*P*‐value = .003) or radiotherapy (*P*‐value = .018). All these patients were more likely to have longer hospital stay; also, the type of reconstruction (one‐ or two‐stage) influenced hospital stay (*P*‐value = .001).

Patients had a mean follow up period of 22.7 months (range 3‐75.7): they were postoperatively checked in each clinic following standard practice and patients’ complications were recorded and treated during follow up visits. Complications were analyzed considering the problem as breast‐related, not patient‐related, as each breast has its own individual risk factors. For example, a patient who underwent a bilateral mastectomy and PPBR may need radiation therapy on one side and no therapy on the contralateral breast, this way risk factors for postoperative complications would differ between the breasts.

As shown in Table 2, a total of 200 breasts experienced immediate complications while late complications occurred in 61 breasts; 46.7% of the total complications had no records about their onset. Among all PPBR, the implant loss rate was 6.5%, while 76.2% of all complicated breasts were conservatively treated. Most of the implant losses occurred in the early period (41 breasts; 2.8% vs 27 breasts; 1.9%); moreover, immediate complications were mainly surgery‐related adverse events such as seroma, wound dehiscence, hematoma, skin necrosis and infection. On the other side, late complications are mostly represented by implant‐related problems, in particular, capsular contracture and rippling (Table 2). In a considerable number of patients, the timing of adverse events was not specified, so the complication timing was not included in the statistical analysis, focusing it on the total complication number and on the risk factors affecting their occurrence.

**Table 2:** Complications, sorted by the timing of occurrence

Complications iBAG cohort: 1186 patients/1450 breasts	Total values		Early complications (within 3 mo)		Late complications (after 3 mo)		Not defined	
	Breast (Nr)(%)	Patients (Nr)(%)	Breast (Nr)(%)	Patients (Nr)(%)	Breast (Nr)(%)	Patients (Nr)(%)	Breast (Nr)(%)	Patients (Nr)(%)
Seroma	111 (7.7)	103 (8.7)	52 (3.6)	47 (4.0)	3 (0.2)	3 (0.3)	56 (3.9)	53 (4.5)
Dehiscence	67 (4.6)	63 (5.3)	28 (1.9)	27 (2.3)	0 (0)	0 (0)	39 (2.7)	36 (3.0)
Hematoma	31 (2.1)	31 (2.6)	20 (1.4)	20 (1.7)	0 (0)	0 (0)	11 (0.8)	11 (0.9)
Necrosis	46 (3.2)	42 (3.5)	21 (1.4)	18 (1.5)	2 (0.1)	2 (0.2)	23 (1.6)	22 (1.9)
Infection	70 (4.8)	64 (5.4)	25 (1.7)	23 (1.9)	5 (0.3)	5 (0.4)	40 (2.8)	36 (3.0)
Extrusion	18 (1.2)	17 (1.4)	6 (0.4)	6 (0.5)	5 (0.3)	5 (0.4)	7 (0.5)	7 (0.6)
RBS	48 (3.3)	46 (3.9)	17 (1.2)	16 (1.3)	1 (0.1)	1 (0.1)	30 (2.1)	29 (2.4)
Fever	25 (1.7)	16 (1.3)	19 (1.3)	12 (1.0)	0 (0)	0 (0)	6 (0.4)	4 (0.3)
Implant rotation	3 (0.2)	3 (0.3)	0 (0)	0 (0)	3 (0.2)	3 (0.3)	0 (0)	0 (0)
Capsular contracture	31 (2.1)	26 (2.2)	5 (0.3)	4 (0.3)	17 (1.2)	14 (1.2)	9 (0.6)	8 (0.7)
Rippling	40 (2.8)	30 (2.5)	7 (0.5)	4 (0.3)	25 (1.7)	19 (1.6)	8 (0.5)	7 (0.6)
Other complications	48 (3.3)	46 (3.9)	11 (0.8)	9 (0.8)	6 (0.4)	6 (0.5)	31 (2.1)	31 (2.6)
Implant loss	94 (6.5)	89 (7.5)	41 (2.8)	37 (3.1)	27 (1.9)	25 (2.1)	26 (1.8)	26 (2.2)

Abbreviation: RBS, red breast syndrome.

Among the breasts that developed complications, most showed the incidence of a single adverse event (n = 284; 72.6%). In these cases, the removal of the implant was carried out in 17.6% (n = 50). Considering breasts suffering from two complications (n = 66; 16.9%), implant loss rate raise at 30.3% (n = 20), while breasts that reported three or more complications (n = 37; 9.5%) led to implant loss in more than half of the cases (n = 22; 59.5%). Two women had the removal of the breast implant but no records on occurred complications were collected.

The multivariate analysis of the risk factors contributing to complication onset underlined an increased risk of seroma formation in patients using immunosuppressive drugs (RR = 4.56; *P*‐value = .017) as well as those who had had a previous esthetic breast surgery (RR = 2.54; *P*‐value = .030) or an axillary dissection during the PPBR (RR = 1.67; *P*‐value = .039); also the type of tumor seemed to increase seroma onset (*P*‐value = .029).

Infection instead is more likely to occur in diabetics (RR = 4.026; *P*‐value = .003) and ex/active smokers (*P*‐value = .016) with RR = 2.15 for ex‐smokers and 2.08 for active smokers. Axillary dissection also influenced infection onset (RR = 2.17; *P*‐value = .009) and an increasing implant volume showed, both in univariate and multivariate analysis, a higher risk of infection development, with an infected breast mean volume of 409.3 g compared to those of noninfected cases of 346.2 g (*P*‐value = .001). Always regarding the infection occurrence, statistical significance was highlighted both by univariate and multivariate analysis for the type of incision used (*P*‐value <.05) except for the round‐block technique.

Considering risk factors for wound dehiscence, multivariate analysis demonstrated a high impact of active smoking habits on this complication (RR = 2.49; *P*‐value = .001). Moreover, skin reducing mastectomy appeared to be more at risk of wound dehiscence (RR = 5.19; *P*‐value = .047) compared to nipple‐sparing mastectomy; also the type of surgical incision chosen (*P*‐value <.05), particularly wise pattern, influenced wound dehiscence.

Smoking status, immunosuppressive drugs and implant volume were risk factors involved in postoperative mastectomy flap necrosis (*P* < .05); particularly, mean implant volume in complicated patients was 406.8 g while for the other ones it was 347.3 g.

Active smokers and diabetics are prone to develop RBS (respectively RR = 2.42, RR = 3.90; *P*‐value = .02); the presence of a previous mastectomy has demonstrated a connection with RBS as well (RR = 6.55; *P*‐value = .044). The univariate analysis also gave significance to the implant volume variable (*P*‐value = .004) although the multivariate did not confirm the value (*P*‐value = .058).

Capsular contracture was related to postoperative radiation therapy (RR = 3.90; *P*‐value < .05) both in univariate and multivariate analysis; moreover, this complication seems to be more common in patients having a PPBR for revision surgery (RR = 16.36; *P*‐value = .001) or having had a previous breast augmentation (RR = 5.02; *P*‐value = .009) or breast reconstruction (RR = 8.93; *P*‐value < .05).

Patients were further divided into two subgroups: those undergone radiation therapies (pre‐ or postoperatively) and those not irradiated, to evaluate with a univariate analysis the potential impact of this treatment on postoperative complications (Table 3). Through univariate analysis, it was investigated also if the administration time of radiation therapy, pre‐ or postoperatively, could represent a significant risk factor for complication development. Radiation therapy appeared to be a statistically significant risk factor for the development of postoperative seroma, capsular contracture, rippling, and implant loss. Interestingly, at multivariate analysis, the negative impact of the postoperative treatment was confirmed only on capsular contracture, having no statistically significant association with the occurrence of all other complications (Table 3).

**Figure 2:**
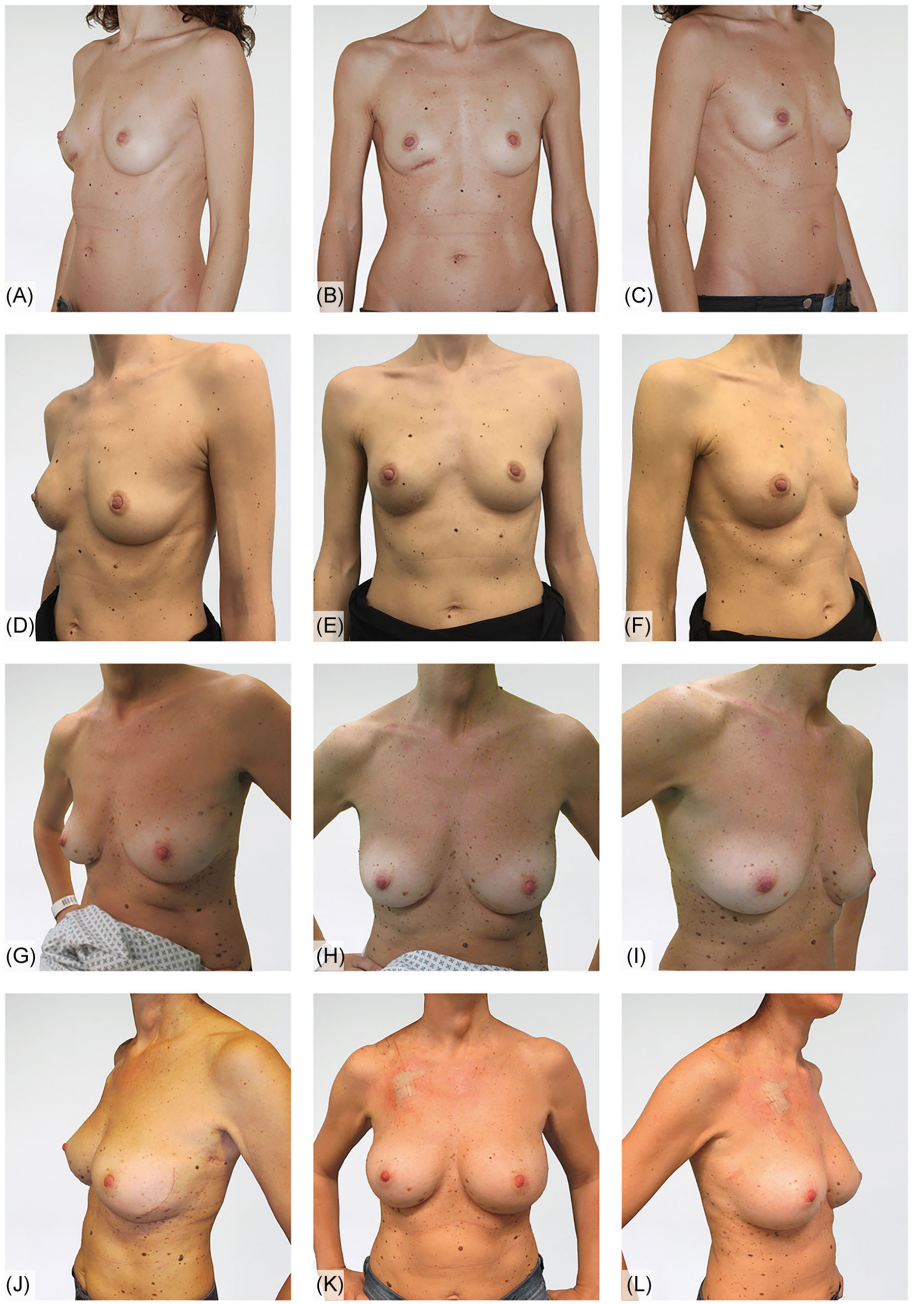
A‐C, Preoperative pictures in lateral and frontal view, slight asymmetry of the right breast due to a previous clinical investigation; D‐F outcomes at 21 months of unilateral right nipple‐sparing mastectomy and prepectoral breast reconstruction with Braxon; G‐I, preoperative lateral and frontal views of an IDC left case; J‐L, outcome of left immediate prepectoral reconstruction after nipple‐sparing mastectomy and right breast augmentation, 7 months postop. IDC, invasive ductal carcinoma [Color figure can be viewed at wileyonlinelibrary.com]

**Figure 3:**
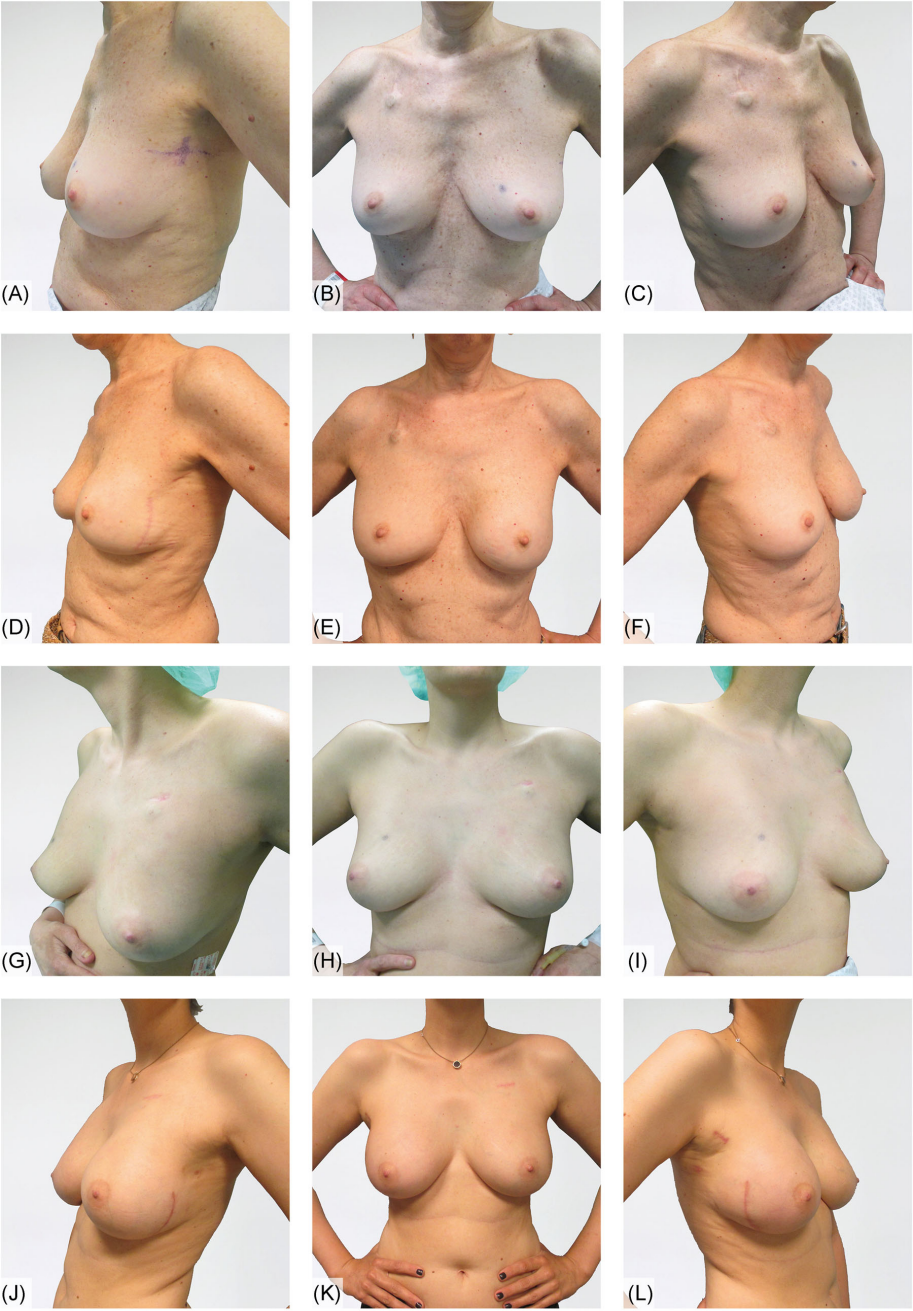
A‐C, Preoperative lateral and frontal views; D‐F, 12 months postop outcomes of left immediate prepectoral breast reconstruction; G‐I, frontal and lateral views of right IDC case; J‐L, 7 months from the bilateral prepectoral reconstruction after nipple‐sparing mastectomies [Color figure can be viewed at wileyonlinelibrary.com]

**Figure 4:**
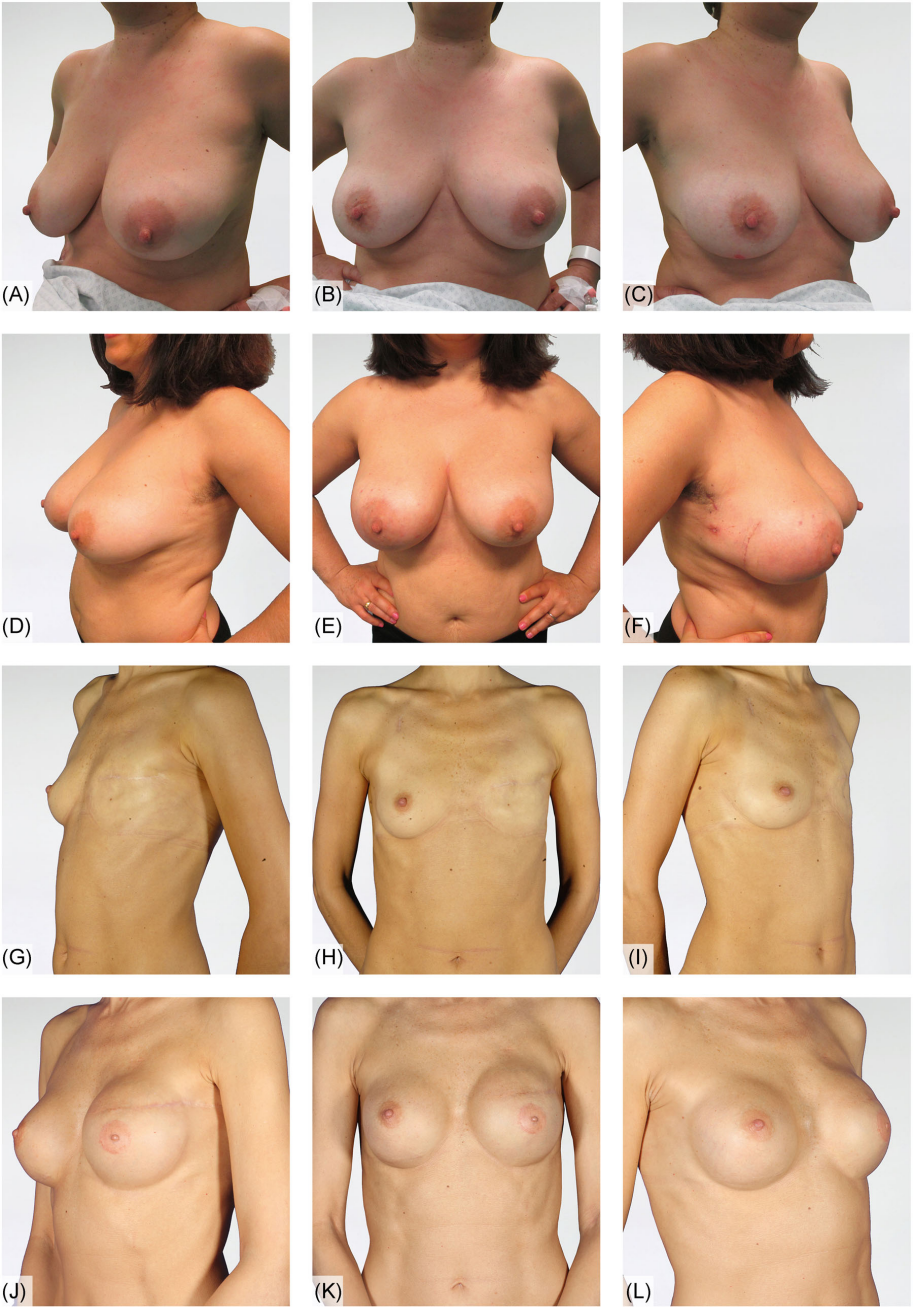
A‐C, Preoperative lateral and frontal pictures of a right DCIS case; D‐F, esthetic result after 6 months of right immediate prepectoral reconstruction; G‐I, preoperative lateral and frontal pictures of a failure of previous left breast reconstruction; J‐l, outcomes of left delayed prepectoral breast reconstruction, 24 months postoperative. DCIS, ductal carcinoma in situ [Color figure can be viewed at wileyonlinelibrary.com]

Six patients are shown in Figures 2, 3, 4 as representative examples of PPBR esthetic outcomes. Each figure shows two cases with preoperative and postoperative pictures in lateral and frontal views.

## 4 DISCUSSION

Prepectoral ADM‐assisted breast reconstruction is growing in fame among all European and American hospitals, with the aim of improving patients’ esthetic and functional outcomes, such as their quality of life.^5^, ^6^ The advantages of PPBR compared to the traditional submuscular approach are well described in several publications, but lack of data from large cohorts of patients negatively affects the quality of scientific information. Moreover, inadequate information about the technique also affects the surgeon's judgment for a correct patient selection, thus increasing the risk of developing postoperative complications. The aim of this data collection was to obtain the largest evidence on PPBR performed with a complete Braxon ADM implant wrapping, to acquire unquestionable statement about PPBR outcomes and consequently lead to correct patient selection.

This retrospective multicentre study collected 1450 cases, thus defining the largest database of ADM‐assisted PPBR patients; complication rates were acceptable and always below 8%, with an overall reconstruction failure of 6.5%. Statistical analysis showed a higher risk of developing postoperative complications for active smokers, diabetics, and patients who had had previous breast surgery or currently taking immunosuppressants; additionally, some surgical details such as implant volume, type of skin incision or mastectomy seem to affect complication rate. The adverse effect of postoperative radiation therapy was confirmed only on capsular contracture occurrence (RR = 3.90; *P*‐value < .05) resulting anyway with a low overall rate, set around 2%.

The few publications about one‐step PPBR reported outcomes in line with the data raising from the iBAG study. Evidence about immediate one‐step PPBR are mainly from European clinical experiences, most of them performed with the use of Braxon ADM.^3^, ^4^, ^5^, ^6^, ^11^, ^12^, ^13^ From these publications a similar rate of implant loss is observed, set around 4%; an exception is represented by Vidya's multicentre study, which reported 2% of failure rate on 100 cases; this series was characterized by highly selected patients, with low associated risk factors, and this could explain the low complication rate.^11^

The study by Reitsamer et al on 200 PPBR with implants wrapped either with ADM or synthetic mesh showed the following complication rate among the total reconstructed breasts in a 36‐months follow‐up: 7% of necrosis, 4% of hematoma, 14.5% of seroma, 2.5% of implant rotation and 1.5% of rippling; lipofilling was required in 3.5% of the women. Although complication rates in this study were quite higher than those reported in the iBAG study, we are not able to associate them specifically with one of the two materials used to cover the implant, since the authors did not correlate outcomes with membrane type.^14^ Instead, we are led to consider the surgical approach chosen as the critical factor impacting outcomes, since 79.5% of cases underwent mastectomy through inframammary fold incision versus 13% of iBAG experience. In fact, this surgical access reduces breast pocket visibility, thus increasing the risk of accidental damage to nipple vascular plexus, thus explaining the higher rate of nipple‐areolar complex necrosis (7% vs 3.2% of the iBAG cohort). Even seroma incidence is doubled in Reitsamer's paper, when compared to 7% of the present study; this could be explained with the presence of chemical preservatives (Polysorbate 20) present in the ADM used, which may cause a higher postoperative inflammatory reaction. An observation should be also made on implant rotation rate, 12 times higher than that reported in iBAG data, which may originate from the different wrapping technique: in fact, Reitsamer's approach involves only a frontal implant covering while iBAG study used an ADM able to create a whole “pocket” which tightly fits the anatomical profile of the breast implant and avoid implant rotation.

In Highton et al's^15^ and Chandarana et al's^12^ experiences, the rate of necrosis was found to be slightly higher than in the iBAG collection, as they reported respectively 4.4% and 5.2% rates. Even though the ADM used was the same as iBAG study, immediate and late complication rates of Chandarana's analysis were consistently higher than ours (hematoma 6.9%, infection 6%, seroma 13.3%). In our opinion, the differences in clinical and surgical details of the populations between the two studies might have played a role in adverse events occurrence.

Overseas, studies have been published by American authors which have performed PPBR on larger cohorts, yet in a two‐stage procedure with the use of tissue expander covered with ADM positioned on the frontal part only.^16^ Moreover, the use of porcine ADM is still uncommon among the main American scientific papers, so that most evidence derives from human dermis such as AlloDerm.^17^ The analysis of these studies should bring up concern on more aspects, the fact that two‐stage techniques require a second surgery to exchange the device with a definitive implant and the relative increased risk of tumor recurrence.^18^ In European daily practice, a direct‐to‐implant single‐stage PPBR is preferably offered, since in most women a second procedure can be easily spared. Moreover, multiple surgeries are associated with an increased infection risk which could find confirmation in higher infection rates reported in the main American PPBR experiences.^19^ In fact, Nahabedian reported very high infection rates (12.8% by patient; 8.1% by breast) not in line with iBAG study outcomes, while implant loss rate was similar (6.5%) and slightly different necrosis and seroma rates (9.7% and 4.8%).^17^ High implant and expander loss rate, as well as infection one, were also found in Bettinger and Schnarrs publications about prepectoral expander/implant reconstructions, with overall infection rates of 9.1% for the first one and 11.8% for the second, reinforcing again our hypothesis.^20^, ^21^

Furthermore, patient's risk factors revealed by our statistical analysis find confirmations in other publications: Schnarrs highlighted higher postoperative complications risk in smokers and patients with >500 g breasts,^20^ while Bettinger observed increased complication rates in previously irradiated breasts and no significant association between chemotherapy and postoperative adverse events.^21^

In iBAG analysis a secondary interest was to investigate RT impact on postoperative outcomes; the multivariate analysis did not reveal any statistically significant correlation between RT and postoperative complications, except for capsular contracture, that even so did not reach a high percentage (about 5%) (Table 3). Very few publications until now have focused their studies on PPBR and RT and moreover their populations were limited to small cohorts with a maximum of 54 patients undergone unilateral RT.^22^, ^23^, ^24^ Even in these series the authors had experienced only with the two‐stage reconstructive procedure and not with a direct‐to‐implant (DTI) PPBR. With regard to the impact of radiotherapy on PPBR outcomes, the iBAG study collected 190 irradiated patients for a total of 198 DTI‐PPBR, which to our knowledge represent the largest evidence on this issue. The outcomes in Table 3 demonstrated, especially for postoperative RT, no association with the onset of postoperative complications, except for capsular contracture.

**Table 3:** Comparison of irradiated and nonirradiated breasts developing postoperative complications^a^

Complications	Nonirradiated Nr = 1243 breasts	Irradiated Nr = 198 breasts^b^			Preop RT Nr = 45 breasts^c^			Postop RT Nr = 159 breasts^c^		
	Nr (%)	Nr (%)	RR	*P*‐value	Nr (%)	RR	*P*‐value	Nr (%)	RR	*P*‐value
Seroma	87 (7.0)	22 (11.1)	1.53	.042	5 (11.1)	1.52	.363	17 (10.7)	1.49	.100
Dehiscence	56 (4.5)	8 (4.0)	0.91	.768	1 (2.2)	0.49	.461	7 (4.4)	1.02	.951
Hematoma	27 (2.2)	4 (2.0)	0.97	.948	0 (0.0)	0.00	.320	4 (2.5)	1.23	.662
Necrosis	38 (3.1)	8 (4.0)	1.28	.465	2 (4.4)	1.41	.630	6 (3.8)	1.21	.632
Infection	59 (4.7)	10 (5.1)	1.06	.852	2 (4.4)	0.92	.910	8 (5.0)	1.09	.810
Extrusion	13 (1.0)	5 (2.5)	2.05	.082	3 (6.7)	5.63	<.05	2 (1.3)	1.08	.906
RBS	44 (3.5)	4 (2.0)	0.60	.268	0 (0.0)	0.00	.205	4 (2.5)	0.76	.565
Fever	24 (1.9)	1 (0.5)	0.29	.153	0 (0.0)	0.00	.364	1 (0.6)	0.36	.265
Implant rotation	3 (0.2)	0 (0.0)	0.00	.489	0 (0.0)	0.00	.755	0 (0.0)	0.71	.545
Capsular contracture	21 (1.7)	10 (5.1)	2.42	.002	0 (0.0)	0.00	.312	10 (6.3)	3.10	<.05
Rippling	39 (3.1)	1 (0.5)	0.18	.036	1 (2.2)	0.79	.817	0 (0.0)	0.00	.025
Other complications	39 (3.1)	9 (4.5)	1.38	.305	2 (4.4)	1.35	.675	7 (4.4)	1.36	.401
Implant Loss	70 (5.6)	22 (11.1)	1.83	.003	5 (13.3)	1.83	.189	17 (10.7)	1.80	.014

*Note*: 2. Nr. 6 breasts had data about preoperative treatment but no evidence about the postoperative therapy; Nr. 2 breasts had data about postoperative radiation but no details about preoperative treatment.

Abbreviation: RBS, red breast syndrome.

a Nr. 9 breasts had no data on radiation therapy.

b Nr. 6 breasts had data about preoperative treatment but no evidences about the postoperative therapy; Nr. 2 breasts had data about postoperative radiation but no details about preoperative treatment.

c Nr. 3 breasts had both treatments, pre and postoperative.

The retrospective review by Elswick et al about irradiated PPBR collected the largest number of patients published up to now but with just 9 months of follow‐up; in this study, RT was found not to have any statistical association with adverse event occurrence, despite the overall higher rates of complications in the irradiated group vs the nonirradiated one (25.9% vs 23.1%): infection rate was high in both groups (18.5% vs 7.7%), as in most American publications on PPBR. Capsular contracture rate was higher in the irradiated group (1.9% vs 0%), as we observed in iBAG experience (5.1% vs 1.7%); the slightly different percentage could be probably due to the different distribution of the iBAG population, which was richer in smokers (7.9%), diabetics (1%) and patients with hypothyroidism (7.1%), when compared to Elswick's review, or due to the shorter follow‐up of this review.^24^

In the same year, the publication by Sinnott et al^25^ compared irradiated and nonirradiated patients, stratified by prepectoral or subpectoral procedure; the subpectoral reconstruction showed overall higher capsular contracture rate than PPBR (9.8% vs 5.2%), especially in irradiated breasts (52.2% vs 16.1%), confirming the ability of ADM to prevent from capsular contracture and the effectiveness of prepectoral implant positioning vs the submuscular one.

Prepectoral and submuscular breast reconstruction were also evaluated by Sbitany and colleagues comparing irradiated and not‐irradiated breasts from a single center experience; the two groups undergoing postmastectomy RT revealed different complication rates with overall higher rates in PPBR group. Infection represented again the highest issue (42.4% in PPBR vs 32.2%), followed by explant rate (15.4% in PPBR vs 19.3%), wound dehiscence (15.4% in PPBR vs 12.9%), skin necrosis (26.9%, in PPBR vs 9.7%), seroma and hematoma (both 7.7%. in PPBR vs 3.2%). Despite the high incidence of complications, no statistical evidence of influence between postmastectomy RT and PPBR outcomes were observed.^26^

The role of ADM in the prevention of periprosthetic fibrosis was evaluated in several papers, also before the conception of PPBR, and its positive effect was confirmed in mutual agreement.^27^, ^28^ Moreover, the study by Onesti et al^4^ described the specific behavior of the ADM Braxon confirming, through ultrasound and histologic examinations, the formation of a new layer in the subcutaneous tissue that is less prone to contract during the years. Nevertheless, in some recent publications on PPBR, the ADM implant covering has been questioned again. The study by Salibian et al^29^ proposed a staged subcutaneous reconstruction without any expander cover, but it revealed 7.6% of capsular contracture (Baker III and IV) in a series of 155 patients (250 breasts) reconstructed with a “naked” prepectoral expander. The same doubt about the need of ADM to cover the implant in PPBR was the basis of the retrospective study by Singla et al^30^ from the analysis of the immediate single‐stage subcutaneous reconstructions carried out without any coverage of the silicone implant raised the following complications rate: 15.3% of seroma, 15.3% of infections, 11.5% of implant rotation, 3.8% of capsular contracture and 19.2% of implant visibility and contour defects; far from the results of iBAG data collection. These results lead us to consider the complete ADM cover a crucial point in PPBR to reduce the risk of periprosthetic fibrosis.

In conclusion, the iBAG data collection represents the largest study on PPBR performed using a complete implant wrapping with a preshaped porcine ADM. Data from this retrospective multicentre audit confirm the effectiveness of the technique and the low postoperative complication rate, especially capsular contracture; moreover, an ideal patient profile has been drawn by assessing the risk factors involved in postoperative complications’ onset.

The authors agree to consider this study limited by its retrospective nature, especially for those patient's characteristics and intraoperative details that were collected in a percentage too small to reach the value sufficient for the statistical analysis and which in the future should be further investigated to better describe the correct patient selection. For this reason, a prospective study is planned: starting from the outcomes of this large data collection on PPBR we will analyze the impact of a more careful patient selection on the postoperative complication as well as implant loss rate. A prospective evaluation of the effect of radiotherapy is planned too. Other limits of this study were that esthetic and patient‐reported outcomes were not reported and that postoperative pain scores were available only for a minority of patients so that could not be included as an outcome measure. This leaves room for further discussion of results, in particular in a longer follow‐up and a greater number of irradiated patients so that to draw more stable conclusions in this interesting subgroup of patients.

## 5 CONCLUSIONS

The iBAG study brings the largest evidence on PPBR, collecting the experience of 30 hospital centers in a 6‐year period. The audit collected homogeneous data on 1450 procedures carried out by wrapping the implant with a preshaped porcine ADM; data analysis highlighted lasting results with low complication rates and defined the risk factors involved in the onset of postoperative complications. The iBAG multicentre data collection represents a milestone in the field of breast reconstruction, extensively improving the knowledge of this procedure. Further prospective studies are expected to achieve a clearer picture of a patient's satisfaction and the impact of radiotherapy on the outcomes of prepectoral reconstruction.

## iBAG WORKING GROUP

Marzia Salgarello, MD, Department of Plastic Surgery and Reconstructive Surgery, Fondazione Policlinico Universitario Agostino Gemelli IRCCS, Università Cattolica del Sacro Cuore (Rome – IT). Leonardo Cattelani, MD, Breast Surgery Unit, University Hospital of Parma (Parma – IT). Pier Camillo Parodi, MD, PhD, Department of Plastic and Reconstructive Surgery, Azienda Ospedaliero‐Universitaria “Santa Maria della Misericordia” (Udine – IT). Diego Ribuffo MD, Plastic Surgery Unit, Department of Surgery "P. Valdoni", Sapienza University (Rome – IT). Maria Giuseppina Onesti, MD, Plastic Surgery Unit, Department of Surgery "P. Valdoni", Sapienza University (Rome – IT). Giorgio Berna MD, Plastic and Reconstructive Surgery Department, “Ca’ Foncello” General Hospital, AULSS2 Marca Trevigiana (Treviso – IT). Simon J. Cawthorn MD, Southmead Hospital Bristol, North Bristol NHS Trust (Bristol – UK). Fernando Bozza MD, Breast Care Center, IOV Istituto Oncologico Veneto ‐ IRCCS (Padova – IT). Stefano Duodeci MD, Breast Unit, Hospital of Cittadella, AULSS6 Euganea (Padova – IT). Simon Harries MD, Warwick Hospital, South Warwickshire NHS Foundation Trust (Warwick – UK). Maurizio Governa MD, Department of Plastic and Reconstructive Surgery, Azienda Ospedaliera Universitaria Integrata (Verona – IT).Roberto A. Barmasse MD, Unit of Thoracic and Breast Surgery, “Umberto Parini” General Hospital (Aosta – IT). Raghavan Vidya MD, New Cross Hospital, Royal Wolverhampton NHS Trust (Wolverhampton – UK). Tapan Sircar MD, New Cross Hospital, Royal Wolverhampton NHS Trust (Wolverhampton – UK). Harleen Deol MD, East and North Hertfordshire NHS Trust (Welwyn Garden City – UK). Tommaso Guzzetti MD, Plastic Surgery Unit, ASST "Alessandro Manzoni" Hospital (Lecco – IT). Fabrizio Meggiolaro MD, Breast Unit, Mirano Hospital, AUSSL3 Serenissima (Venezia – IT). Mauro Schiavon MD, Department of Plastic and Reconstructive Surgery, Azienda Ospedaliero‐Universitaria “Santa Maria della Misericordia” (Udine – IT). Rishikesh Parmeshwar MD, Department of Breast and General Surgery, University Hospitals of Morecambe Bay NHS Foundation Trust (Lancaster – UK). Douglas Ferguson MD, Royal Devon and Exeter Hospital, Royal Devon and Exeter NHS Foundation Trust (Exeter – UK). Caroline Mortimer MD, Ipswich Hospital, East Suffolk and North Essex NHS Foundation Trust (Ipswich – UK). Giorgio Manca MD, Department of Plastic Surgery, Spedali Civili Brescia, University of Brescia (Brescia – IT). Dinesh Thekkinkattil MD, Department of Oncoplastic Breast Surgery, Pilgrim Hospital and Lincoln County Hospital, United Lincolnshire Hospitals NHS Trust (Lincoln – UK). Rathinasabapathy Rathinaezhil MD, Park Centre for Breast Care, Brighton and Sussex University Hospitals NHS Trust (Brighton – UK). Pud Bhaskar MD, University Hospital of North Tees, North Tees and Hartlepool NHS Foundation Trust (Stockton‐on‐Tees – UK). Nicola Roche MD, The Royal Marsden Hospital, The Royal Marsden NHS Foundation Trust (Chelsea, London – UK). Alberto Rivarossa MD, Department of Plastic and Reconstructive Surgery, “Santa Croce e Carle” Hospital (Cuneo – IT). Gonzalo De Castro Parga MD, PhD, Breast Surgery, University Hospital Complex of Vigo CHUVI (Vigo ‐ ES). Samuele Massarut MD, Breast Surgery Unit, CRO National Cancer Institute (Aviano – IT). Susanna Polotto MD, Breast Surgery Unit, University Hospital of Parma (Parma – IT). Mattia Di Bartolomeo MD, Department of Plastic Surgery and Reconstructive Surgery, Fondazione Policlinico Universitario Agostino Gemelli IRCCS, Università Cattolica del Sacro Cuore (Rome – IT). Emanuele Rampino Cordaro MD, Department of Plastic and Reconstructive Surgery, Azienda Ospedaliero‐Universitaria “Santa Maria della Misericordia” (Udine – IT). Sebastiano Mura MD, Department of Plastic and Reconstructive Surgery, Azienda Ospedaliero‐Universitaria “Santa Maria della Misericordia” (Udine – IT). Liliana Barone Adesi MD, Department of Plastic Surgery and Reconstructive Surgery, Fondazione Policlinico Universitario Agostino Gemelli IRCCS, Università Cattolica del Sacro Cuore (Rome – IT). Giovanni Marruzzo MD, Plastic Surgery Unit, Department of Surgery “P. Valdoni”, Sapienza University (Rome – IT). Francesco Dell'Antonia MD, Plastic and Reconstructive Surgery Department, “Ca’ Foncello” General Hospital, AULSS2 Marca Trevigiana (Treviso – IT). Monia Maritan MD, Plastic and Reconstructive Surgery Department, “Ca’ Foncello” General Hospital, AULSS2 Marca Trevigiana (Treviso – IT). Tania Saibene MD, Breast Care Center, IOV Istituto Oncologico Veneto ‐ IRCCS (Padova – IT). Silvia Michieletto MD, Breast Care Center, IOV Istituto Oncologico Veneto ‐ IRCCS (Padova – IT). Claudia Cecconi MD, Breast Care Center, IOV Istituto Oncologico Veneto ‐ IRCCS (Padova – IT). Manuele Maino MD, Breast Unit, Hospital of Cittadella, AULSS6 Euganea (Padova – IT). Dayalan Clarke MD, Warwick Hospital, South Warwickshire NHS Foundation Trust (Warwick – UK). Sara Mirandola MD, Department of Plastic and Reconstructive Surgery, Azienda Ospedaliera Universitaria Integrata (Verona – IT). Lucia Morelli MD, Unit of Thoracic and Breast Surgery, “Umberto Parini” General Hospital (Aosta – IT). Mario Biral MD, Breast Unit, Mirano Hospital, AUSSL3 Serenissima (Venezia – IT). Roberto Baraziol MD, Department of Plastic and Reconstructive Surgery, Azienda Ospedaliero‐Universitaria “Santa Maria della Misericordia” (Udine – IT). Chiara Zanin MD, Department of Plastic and Reconstructive Surgery, Azienda Ospedaliero‐Universitaria “Santa Maria della Misericordia” (Udine – IT). Chiara Gregorelli MD, Department of Plastic Surgery, Spedali Civili Brescia, University of Brescia (Brescia – IT).

## CONFLICT OF INTERESTS

The authors declare that there are no conflict of interests.

## AUTHOR CONTRIBUTIONS

All the participant to the iBAG study must be considered author of the study. They all had substantial contributions to the design of work, drafted work, made the final approval, and agreed to be accountable for all aspects of the work.

## SYNOPSIS

The authors performed the largest retrospective data collection on PPBR with the Braxon ADM among 30 European and UK centers, collecting a total of 1450 reconstructed breasts between November 2012 and June 2018. The analysis revealed low overall complication rates and the risk factors associated with their occurrence, setting a milestone in the history of breast reconstruction.

## Data Availability

The data that support the findings of this study are available from the corresponding author upon reasonable request.
